# Concomitant chronic subdural hematomas and arachnoid cysts in young adults

**DOI:** 10.12688/f1000research.53210.2

**Published:** 2022-02-07

**Authors:** Huseyin Berk Benek, Emrah Akcay

**Affiliations:** 1 Neurosurgery, University of Health Sciences Izmir Bozyaka Education and Research Hospital, Izmir, 35360, Turkey

**Keywords:** Arachnoid cyst, chronic subdural hematoma, headache, middle fossa, young adult

## Abstract

**Objective: **This study aimed to evaluate the correlation between arachnoid cysts and chronic subdural hematomas in young adults.

**Methods: **This retrospective study evaluated ten patients having concomitant chronic subdural hematomas and arachnoid cysts. Patients were evaluated with the data of age and gender, location of hematoma and arachnoid cyst, trauma history, symptoms at admission, maximum hematoma diameter, contiguity between arachnoid cyst and hematoma, and treatment  methods.

**Results:** We treated 285 patients who were diagnosed with cSDH between January 2013 and December 2019. 22 patients were under the age of 40 years. Ten of them had both cSDH and arachnoid cysts. The mean age of patients was 24.8±3.9 years. Patients with only chronic subdural hematoma had higher mean age than the patients with arachnoid cyst-related chronic subdural hematoma. In four patients, the onset of chronic subdural hematoma was reported after arachnoid cyst diagnosis. Four of the patients did not have causative trauma history, and two patients suffered minor sports-related traumas. All patients had headache, and only two patients had hemiparesis. The location of arachnoid cysts were in the middle fossa in eight patients. All patients had chronic subdural hematomas on the ipsilateral side of arachnoid cyst. Four patients who had smaller than 10 mm maximal cSDH diameter underwent conservative management. They were followed by serial neuroimaging studies and it was noted that the hematoma disappered and the size of the arachnoid cysts decreased over time without any neurological complication. In six cases, craniotomy was required, and all recovered completely. cSDH did not recur during 5–60 months of follow-up period (median 12 months).

**Conclusions:** It seems that presence of an arachnoid cyst in young adults is a predisposing factor for the formation of chronic subdural hematoma. Coincidentally diagnosed arachnoid cyst patients may be followed up with periodical clinical examinations and neuroimaging studies.

## Abbreviations

AC: arachnoid cyst

cSDH: chronic subdural hematoma

CSF: cerebrospinal fluid

CT: computed tomography

MRI: magnetic resonance imaging

## Introduction

Arachnoid cysts (ACs) are benign, intracranial extra-parenchymal cavities with a prevalence of 0.7% to 1,7% of the population. ^1^^,^^2^ They are commonly observed in the middle fossa, mainly on the left side. ^3^^,^^4^ Chronic subdural hematoma (cSDH) is a complex disease with an overall incidence of 1.7-20.6 per 100,000 persons per year and is more commonly encountered in the elderly population. ^5^ Most ACs are asymptomatic, but they could become clinically obvious if the cyst grows and causes a cerebral parenchyma mass. ^1^^,^^6^ cSDH is a rare complication in patients with intracranial AC. ^2^^,^^3^ Approximately 2-4% of the patients admitted for AC were identified with cSDH. ^2^^-^^4^ In this study, we present ten cases and aimed to investigate if ACs could predispose to cSDH in young adults. Although there are several previous manuscripts, our study is one of the most comprehensive assessments of the combination of ACs and cSDH. ^2^^-^^5^^,^^7^^-^^9^ Since there is still a gap of knowledge about this subject today, we tried to contribute to the body of literature.

## Methods

### Patient population

This study retrospectively reviwed the data of the patient files diagnosed with chronic subdural hematoma who were admitted to University of Health Sciences Izmir Bozyaka Education and Research Hospital Department of Neurosurgery between January 2013 and December 2019. Patients having both a chronic subdural hematoma and an arachnoid cysts were determined and included. We also included young adult cSDH patients under the age of 40 years for comparison. Patients under the age of 18 were not included the study. The same neurosurgeons evaluated all of the patients.

### Data collection

The patient data evaluated were age of patients and gender, location of the hematoma and arachnoid cyst, trauma history, symptoms at admission, cSDH maximum diameter, contiguity between AC and cSDH and treatment methods. Patients aged 18–40 years were accepted as young adults. Magnetic resonance images (MRIs) and computed tomographies (CTs) of patients were assessed. The maximum cSDH diameter measurements were performed on axial MRI slices. In six cases, we performed open cranitomy with the evacuation of the hematoma and arachnoid cyst.

### Statistical analysis

Statistical Package for the Social Science 20.0 **(IBM SPSS Statistics, RRID:SCR_019096)** was used for analysis of parameters; JASP ( **RRID:SCR_015823**) is an open access alternative which can perform the same function. Univariate analyses were performed using the Mann–Whitney U-test for non-normally distributed scale parameters. Median and ranges were used for description of scale parameters. All analysis were performed at 95% confidence interval and 0.05 significance level.

## Results

We treated 285 patients who were diagnosed with cSDHs at the University of Health Sciences Izmir Bozyaka Education and Research Hospital Department of Neurosurgery between January 2013 and December 2019. 22 (7.7%) of the 285 cSDH patients were aged under 40 years, and the other 263 patients (92.3 %) were more than 40 years old. 10 (45.5%) of the 22 young adult patients were diagnosed with both AC and cSDH. The mean age of these ten patients was 24.8 ± 3.9 years and the range was 19–36 years. Eight patients (80%) were male and two patients (20%) were female. Patients with both AC and cSDH had lower mean age than the patients having only a cSDH (p < 0.0001).

**Figure 1.**
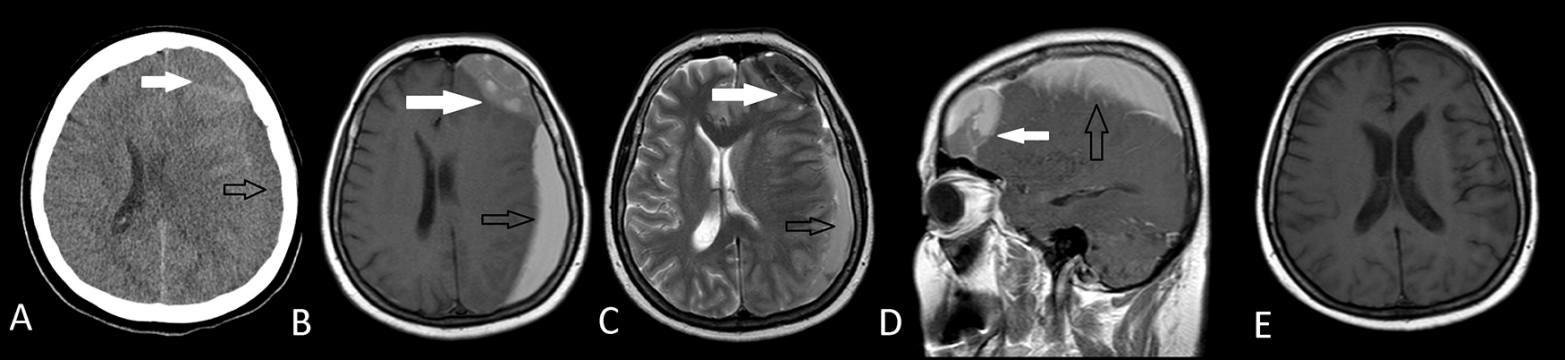
Preoperative axial CT scan (A) and axial T1W MRI (B) axial T2W MRI (C), sagittal MRI (D) images of an arachnoid cyst (white arrows) with ipsilateral subdural hematoma (black arrows) leading to a midline shift in Case 1. Arachnoid cyst had diagnosed before the onset of subdural hematoma and there was no history of a trauma. Postoperative axial T1W MRI (E) shows the total removal of both the arachnoid cyst and the subdural hematoma.

**Figure 2.**
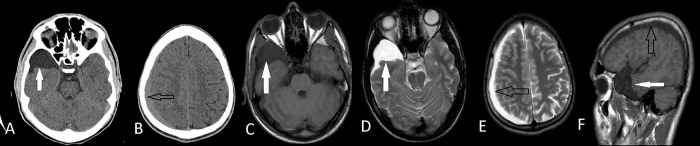
Axial CT images of right middle fossa arachnoid cyst (A) (white arrows) and ipsilateral subdural hematoma (B) (black arrows) of the Case 3. Axial T1W MRI(C), T2W MRI (D,E) and sagittal MRI (F) images shows that they are apart from each other. He had conservative treatment with a complete recovery.

**Figure 3.**
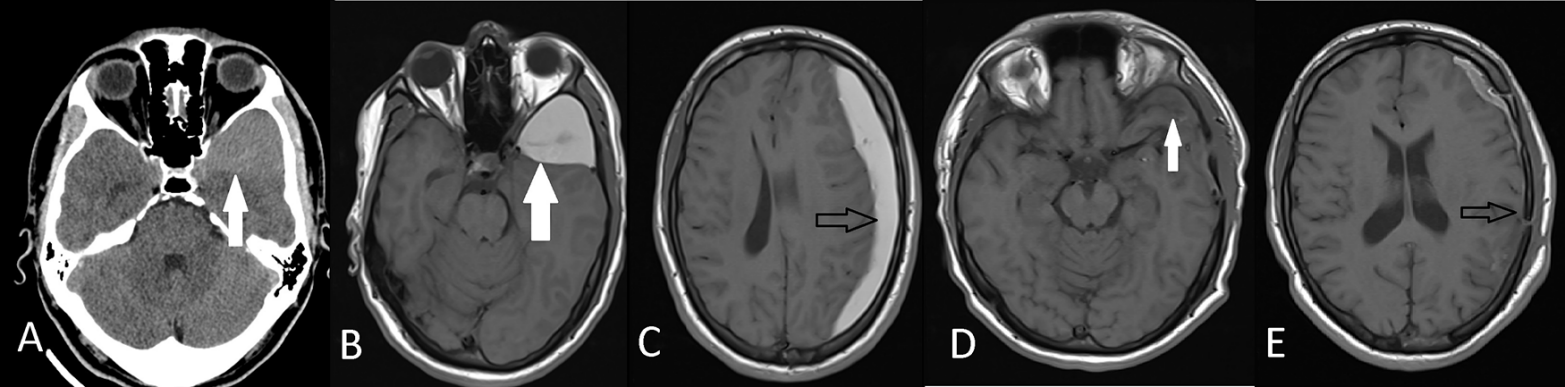
Preoperative axial CT (A) and axial T1W MRI (B) images show left middle fossa arachnoid cyst (white arrows) of Case 6. Axial T1W MRI (C) shows ipsilateral subdural hematoma (black arrows). Postoperative axial T1W MRI (D,E) demonstrates that the hematoma and the arachnoid cyst was evacuated with a tiny fluid in the subdural place.

**Figure 4.**
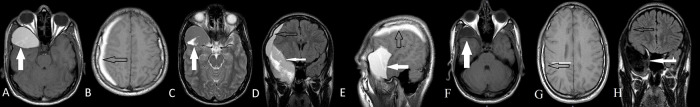
Axial T1W MRI (A,B), T2W (C) MRI, coronal MRI (D) and sagittal MRI (E) images of Case 10 in which a subdural hematoma (black arrows) is close to an arachnoid cyst (white arrows). After two years follow-up, the patient’s AC size decreased as seen on axial T1W MRI (F), and cSDH disappeared thereafter the conservative treatment as seen on axial T1W (G), coronal MRI (H).

The characteristics of the ten patients with AC and concomitant cSDH were shown in the Table 1. In four patients, the onset of cSDH diagnosed after AC diagnosis (Cases 1,5,6 and 9) ( Figures 1, 2, 3). Four patients did not have causative trauma history (Cases 1,3,5,8), two patients sufferred minor sports-related traumas (Cases 2,10). All patients had headache and only two patients had hemiparesis. AC locations were found in the middle fossa in eight patients (Cases 2,3,4,5,6,7,9,10) and two patients had the convexity of the Sylvian fissure location (Cases 1,8). The middle fossa located ACs were Galassi Tip I in seven patients and Galassi Tip II in one patient. ^10^ All patients had cSDHs on the ipsilateral side of AC. None of the patients had cSDH on the contralateral side of AC. In eight patients, cSDH was close to ACs on neuroimaging studies, and the remaining two patients had a cSDH apart from an AC. Surgical intervention was performed in patients with a larger than 10 mm maximal diameter of cSDH. We evacuated the hematoma and arachnoid cyst following open craniotomy in six cases ( Table 1: Cases 1,4,5,6,7,9). We also performed cyst fenestration and tried to remove the cyst membranes as completely as possible. The four patients who had smaller than 10 mm maximal cSDH diameter underwent conservative management ( Table 1: Cases 2,3,8,10). They were followed by serial neuroimaging studies and it was noted that the hematoma disappered and the size of AC decreased over time without any neurological complication ( Figure 4). All six operated patients recovered completely ( Figures 1 and 3). cSDH recurrence was not reported during the period of follow-up of 5-60 months (median 12 months).

**Table 1.** Comparison between young adult patients who had chronic subdural hematomas with and without arachnoid cyst.

Case no	Age/sex	Trauma	Symptoms	AC side/location	SDH side	Maximum SDH diameter (mm)	Contiguity between AC and SDH	Treatment
**1**	36/F	No	Headache	L/frontal- Sylvian	L	27	Y	Craniotomy
**2**	26/M	Minor trauma-ski related	Headache, nausea	L/middle fossa	L	7	Y	Conservative
**3**	19/M	No	Headache	R/middle fossa	R	9	N	Conservative
**4**	24/F	Traffic accident	Headache, vomit, paresis	R/middle fossa	R	12	Y	Craniotomy
**5**	32/M	No	Headache	L/middle fossa	L	22	Y	Craniotomy
**6**	25/M	Fall	Headache	L/middle fossa	L	23	Y	Craniotomy
**7**	21/M	Motorcycle accident	Headache, nausea, paresis	L/middle fossa	L	14	Y	Craniotomy
**8**	20/M	No	Headache	R/Sylvian	R	8	N	Conservative
**9**	24/M	Fall	Headache	L/middle fossa	L	21	Y	Craniotomy
**10**	21/M	Minor trauma—football related	Headache	R/middle fossa	R	8	Y	Conservative

Table 2 shows the comparison of the young adult patients who were between 18–40 years old and had chronic subdural hematomas; ten patients having arachnoid cysts and the twelve patients without arachnoid cysts. The gender, frequency of trauma history, frequency of sports-related trauma, headache incidence, paresis incidence and outcomes distribution differences between patient groups were statistically insignificant (p > 0.05).

**Table 2.** Clinical features and neuroimaging study findings in 10 patients with arachnoid cysts and concomitant chronic subdural hematomas.

Variable	Patients with arachnoid cyst (n = 10)	Patients without arachnoid cyst (n = 12)	p value
**Age (median, years)**	24.8 ± 3.9	27.5 ± 5.9	0.334
**Age ≤21 years**	4 (40%)	1 (8%)	0.054
**Gender, male**	8 (80%)	9 (75%)	1
**Trauma history**	6 (60%)	7 (58%)	1
**Sports-related**	2 (20%)	2 (17%)	1
**Headache**	10 (100%)	12 (100%)	1
**Paresis**	2 (20%)	2 (17%)	1
**Good recovery at discharge**	10 (100%)	12 (100%)	1

## Discussion

In this study, we present ten cases and aimed to investigate the correlation between the chronic subdural hematomas and the arachnoid cysts in young adults. We tried to contribute to the body of literature as there is still a lack of information on this subject today. Subdural hematomas are infrequently encountered complications of arachnoid cysts. Most of the studies about this subject in the literature analyzed the complicated ACs, from a total of patients with cSDH. Parsch *et al.* ^2^ identified AC in 16 of 658 patients (2.4%) with cSDH. Wester and Helland ^3^ reported that 4.6% (11 of 241) of their patients admitted for AC were identified with cSDH. Mori *et al.* ^4^ reported an incidence of 2.2% (12 of 541) of AC in the cases with cSDH. In the case series of Wu *et al.* ^9^ the incidence of AC-associated cSDH was 1.9% (5 of 266). cSDHs are the pathologies that often seen in elderly patients, but uncommonly in young patients. ^11^ When we compared the patients at the same period in our clinic, AC and concomitant cSDH patients had lower age mean than the patients with only cSDH In our study, all ten patients were under age of 36. In our present study, which included 285 cSDH patients, 22 patients (7.7%) were under the age of 40 years. Previously reported rates were 2.4–8.8% and were consistent with the rate of this study. ^6^^,^^8^ 10 young adult patients under the age of 40 years with chronic subdural hematoma were found to have arachnoid cysts (45%). Approximately half of the 18–40-year-old patients had cSDH with AC. Considering that the prevalence of arachnoid cyst is 0.7% to 1.7% of the population, ^2^^,^^3^ we can conclude that ACs may cause cSDH in young patients with or without trauma.

But what is the mechanism of that? Various previous researches have conferred the risk of cSDH related with ACs. ^3^^,^^4^^,^^7^^,^^8^^,^^12^^,^^13^ The exact mechanism is still unclear. However, theories suggest that 1) veins within the wall could be hurt because of decreased compliance 2) veins without structural support of cyst wall are vulnerable 3) a slit-valve mechanism is formed, lead to increased pressure within the AC and vein rupture. ^9^^,^^12^^-^^14^ The compliance of the cyst is less than the normal brain. An increase in ICP causes rupture of these bridging veins. AC-related symptoms usually start with the cyst enlargement. ^9^^,^^12^ In our study, in four patients without evident trauma, these theories explaining the spontaneous rupture of the AC into subdural space may be valid.

According to the ACs and cSDHs locations on neuroimaging studies; three types were defined: 1) a close cSDH to AC, 2) a separate cSDH from an AC on the ipsilateral side, 3) a contralateral side located cSDH to an AC. ^9^^,^^13^ Wester *et al.* ^3^ observed that there are small bridging veins between the dura mater and the outer membrane of the AC. They propounded that the bridging Sylvian veins may cause blood leakage into the subdural place. Rupture of an AC outer wall after head trauma is suggested to cause subdural effusion that could enlarge the cSDH. ^12^^,^^17^ Especially in young adults, spontaneous tearing of the AC wall leads to leakage of CSF and blood into the subdural space. ^4^ Page *et al.* ^12^ declared that ACs are less flexible than the normal without AC brain with reduced intracranial buffering after trauma. ^3^^,^^12^ Thus, hematoma could grow up on the ipsilateral hemispheric subdural space other than AC. The subdural hematoma in Cases 3 and 8 can be explained by this mechanism. The association between cSDH and AC could not be insidental. None of our cases had cSDH on the contralateral side of AC. In previous series, almost half of the 18–40-year-old patients with cSDHs had ACs. ^14^^,^^15^ Young adults with arachnoid cysts tends to be more susceptible to the development chronic subdural hematomas.

Headache is one of the most common syptoms in the patients having both cSDH and AC. In our study, headache was observed in all patients and paresis was observed in two patients who were related with an accident. Headache may be due to increased intracranial pressure. ^18^^,^^19^ Since the subarachnoid space is smaller in young patients than in the elderly, it is possible that they are more affected by increased intracranial pressure, in cSDH enlargement cases. In our study, sports-related cSDH was found in two of the young patients with ACs. Several case reports of cSDH associated with ACs have been reported after head injury in sports. ^20^^-^^23^ Sports is an important factor of cSDHs in young patients. Mori *et al.* ^4^ stated in a study that AC is a risk factor for chronic subdural hematoma in juveniles.

The surgical management of a cSDH related with an AC is a debated subject. Open craniotomy including the membranes of the AC and the cSDH removement, drainage of the hematoma using a burr hole, cyst fenestration or cystoperitoneal shunt could be chosen. The most common surgical method is open craniotomy alone. ^13^^,^^15^ Although some of the recent studies have argued against burr hole irrigation method, we prefered to remove part of the membranes with open craniotomy and to perform cyst fenestration as a safe procedure. Complex dissection interventions could be necessary in such cases in which a burr hole is insufficient in the management. A single burr-hole-mini craniotomy and hematoma evacuation followed by endoscopic inspection of the surgical cavity could be a preferred choise. Initial observation may be considered in patients with a thickness of 10 mm or less cSDH diameter without symtoms of intracranial hypertension. Middle meningeal artery embolization may be represented as a minimally-invazive alternative to surgery for new or recurrent chronic subdural hematomas, or as prophylaxis to reduce the risk of recurrence after surgery. ^24^^,^^25^ Some studies suggest that corticosteroids might be beneficial in the treatment of cSDH; however, there is a lack of well-designed trials that support or refute the use of corticosteroids in cSDH. ^26^ Recurrence can occur in 10%-20% of patients and is associated with several clinical and radiographic predictors. Postoperative cSDH volume and the Nakaguchi classification subtypes proved the most powerful predictors of recurrence and cure (separated or trabecular subtypes and postoperative cSDH volume ≥35.0 mL). ^27^

## Conclusion

It seems to be likely that presence of an arachnoid cyst in young adults is a predisposing factor for the formation of a chronic subdural hematoma with or without a head trauma. Headache is one of the most common symptom in the patients with both arachnoid cyst and chronic subdural hematoma. Coincidentally diagnosed arachnoid cyst patients must be followed up with periodical clinical examinations and neuroimaging studies. Young adults with an arachnoid cyst should be informed of the potential risk of developing a chronic subdural hematoma with forced physical exercises or even spontaneously.

## Ethical approval

This study was approved by the institutional ethics review committee at the University of Helth Sciences Izmir Bozyaka Education and Research Hospital (Date: 13,01,2021, Issue No: 07) in accordance with the World Medical Association Declaration of Helsinki and its most recent amendments. Formal consent was not obligatory for this research.

## Data avaibility

### Underlying data

Open Science Framework: Underlying data for ‘Concomitant chronic subdural hematomas and arachnoid cysts in young adults’. https://doi.org/10.17605/OSF.IO/XM3GW.

Data are available under the terms of the Creative Commons Zero “No rights reserved” data waiver (CC0 1.0 Public domain dedication).
