# Supplementary material for: Concomitant chronic subdural hematomas and arachnoid cysts in young adults
Source: F1000Res. 2022 Apr 28;10:421. Originally published 2021 May 26. [Version 3] doi: 10.12688/f1000research.53210.3 (PMC9226705; doi:10.12688/f1000research.53210.3)
Supplement: Peer review history

# Peer review: Reviewer response for version 3

**DOI:** 10.5256/f1000research.133503.r136266

**Published:** 2022-06-23

**Authors:** Harsh Deora

**Affiliations:** 1 Department of Neurosurgery, National Institute of Mental Health and Neurosciences, Bangalore, Karnataka, India

Acceptable from my end.

Is the work clearly and accurately presented and does it cite the current literature?

Partly

If applicable, is the statistical analysis and its interpretation appropriate?

Yes

Are all the source data underlying the results available to ensure full reproducibility?

No

Is the study design appropriate and is the work technically sound?

Yes

Are the conclusions drawn adequately supported by the results?

Yes

Are sufficient details of methods and analysis provided to allow replication by others?

Yes

Reviewer Expertise:

Neurosurgery

I confirm that I have read this submission and believe that I have an appropriate level of expertise to confirm that it is of an acceptable scientific standard.

# Peer review: Reviewer response for version 3

**DOI:** 10.5256/f1000research.133503.r136267

**Published:** 2022-05-27

**Authors:** Jehuda Soleman

**Affiliations:** 1 Department of Neurosurgery, University Hospital of Basel, Basel, Switzerland

I want to thank the authors for their efforts.

 However, none of my comments have been addressed and taken into account. In my opinion, the study itself does not add valuable information to the existing literature. The conclusions are not based on firm evidence (as mentioned in my previous report concerning the treatment technique: open craniotomy can often be avoided nowadays thanks to endoscopic techniques, while often only the cSDH can be addressed and the cyst left alone; and concerning sports activities in association with AC). In my opinion, the authors did not revise the manuscript accordingly, and therefore, I do not recommend indexing this manuscript.

Is the work clearly and accurately presented and does it cite the current literature?

No

If applicable, is the statistical analysis and its interpretation appropriate?

Partly

Are all the source data underlying the results available to ensure full reproducibility?

No

Is the study design appropriate and is the work technically sound?

No

Are the conclusions drawn adequately supported by the results?

No

Are sufficient details of methods and analysis provided to allow replication by others?

Yes

Reviewer Expertise:

Neurosurgery, Pediatric Neurosurgery, Endoscopy, Vaskular Neurosurgery

I confirm that I have read this submission and believe that I have an appropriate level of expertise to state that I do not consider it to be of an acceptable scientific standard, for reasons outlined above.

# Peer review: Reviewer response for version 2

**DOI:** 10.5256/f1000research.120436.r127929

**Published:** 2022-04-21

**Authors:** Harsh Deora

**Affiliations:** 1 Department of Neurosurgery, National Institute of Mental Health and Neurosciences, Bangalore, Karnataka, India

In this retrospective study, the authors reported ten patients having concomitant chronic subdural hematomas and arachnoid cysts. Subdural hematomas are infrequently encountered complications of arachnoid cysts and the authors have chosen a niche area:

1. Compare Adult, young and pediatric chronic subdural in a tabular form. A useful example: (Deora *et al. *(2021 ^1^)).
2. Discuss how chronic SDH in the arachnoid cysts is different from those due to other reasons like shunts. 
3. Relevant literature review is missing. Arachnoid cysts or arachnoid defects in the spine also lead to chronic SDH. This can also be elaborated upon

If a relevant literature review is done the article is worth accepting from my end.

Is the work clearly and accurately presented and does it cite the current literature?

Partly

If applicable, is the statistical analysis and its interpretation appropriate?

Yes

Are all the source data underlying the results available to ensure full reproducibility?

No

Is the study design appropriate and is the work technically sound?

Yes

Are the conclusions drawn adequately supported by the results?

Yes

Are sufficient details of methods and analysis provided to allow replication by others?

Yes

Reviewer Expertise:

Neurosurgery

I confirm that I have read this submission and believe that I have an appropriate level of expertise to confirm that it is of an acceptable scientific standard, however I have significant reservations, as outlined above.

## References

[1] : Paediatric chronic subdural haematoma: what are the predisposing factors and outcomes in management of these cases?. *Childs Nerv Syst* .38(1) : 10.1007/s00381-021-05341-2 123-132 10.1007/s00381-021-05341-2 34487204

# Response

**Published:** 2022-04-22

**Authors:** Huseyin Berk Benek

**Affiliations:** Bozyaka Training and Research Hospital, Turkey

We report a series of ten patients with concomitant chronic subdural hematomas and arachnoid cysts.

- *Compare Adult, young and pediatric chronic subdural in a tabular form. A useful example: Deora et al. (2021 ^1^)).*
- **Response: **We followed the suggestions and used the mentioned paper. Chronic subdural hematomas (cSDH) are the pathologies that are often seen in elderly patients, but uncommonly in young patients. Incidence in pediatric population is relatively rare. Patients having both a cSDH and an arachnoid cyst (AC) were determined in this study (10 patients, range:18-36 years). Young adults with ACs tend to be more susceptible to the development cSDH. Patients under the age of 18 were not included the study, since pediatric patients were not admitted to our hospital (another center for children). Table 2 shows the comparison of the young adult patients who were between 18–40 years old and had chronic subdural hematomas; ten patients having arachnoid cysts and the twelve patients without arachnoid cysts.
- *Discuss how chronic SDH in the arachnoid cysts is different from those due to other reasons like shunts.*
- **Response:** We discussed the reasons for cSDH (as due to arachnoid cysts or other reasons like shunts). The common predisposing factors of cSDH are diffuse brain atrophy and anticoagulant drugs in elderly population, but these factors are rare in pediatric population. cSDH is a rare complication in patients with intracranial AC. An increase in ICP could cause rupture of these bridging veins. The veins within the wall could be hurt because of decreased compliance. Rupture of an AC outer wall after head trauma is suggested to cause subdural effusion that could enlarge the cSDH. Especially in young adults, spontaneous tearing of the AC wall leads to leakage of CSF and blood into the subdural space. However, over drainage in patients with shunt operations could cause cSDH, the most common reason in pediatric patients.
- Relevant literature review is missing. Arachnoid cysts or arachnoid defects in the spine also lead to chronic SDH. This can also be elaborated upon.
- **Response:** We provided information about arachnoid cysts or arachnoid defects in the spine that cause cSDH. The relevant literature was enlarged with recent articles.
  - [12] Huang KC, Chuang MT, Huang DW, Fang WK, Chen SH, Lee JS. Lumbar intraspinal arachnoid cyst superimposed by hyperacute spinal subdural hematoma: an unusual case. Spine J. 11(7) (2011), e5-8.
  - [13] Tinois J, Bretonnier M, Proisy M, Morandi X, Riffaud L. Ruptured intracranial arachnoid cysts in the subdural space: evaluation of subduro-peritoneal shunts in a pediatric population. Childs Nerv Syst. 36(9) (2020), 2073-2078
  - [14] Deora H, Mishra A, Gupta R, Konar S, Vazhayil V, Shashidhar A, Dwarakanath S. Paediatric chronic subdural haematoma: what are the predisposing factors and outcomes in management of these cases? Childs Nerv Syst. 38(1) (2022),123-132

# Peer review: Reviewer response for version 2

**DOI:** 10.5256/f1000research.120436.r122651

**Published:** 2022-03-10

**Authors:** Roberto Colasanti

**Affiliations:** 1 Department of Neurosurgery, Azienda Ospedali Riuniti, Università Politecnica delle Marche, Ancona, Italy; 2 Department of Neurosurgery, Padua University Hospital, Padova, Italy

The authors replied to my comments. I believe the paper is improved.

Is the work clearly and accurately presented and does it cite the current literature?

Partly

If applicable, is the statistical analysis and its interpretation appropriate?

Partly

Are all the source data underlying the results available to ensure full reproducibility?

Yes

Is the study design appropriate and is the work technically sound?

Partly

Are the conclusions drawn adequately supported by the results?

Partly

Are sufficient details of methods and analysis provided to allow replication by others?

Yes

Reviewer Expertise:

Neurosurgery

I confirm that I have read this submission and believe that I have an appropriate level of expertise to confirm that it is of an acceptable scientific standard.

# Peer review: Reviewer response for version 1

**DOI:** 10.5256/f1000research.56569.r98792

**Published:** 2021-12-13

**Authors:** Jehuda Soleman

**Affiliations:** 1 Department of Neurosurgery, University Hospital of Basel, Basel, Switzerland

Thank you for allowing me to review this manuscript which I read with interest.

- Although the authors claim they add to the literature with their 10 cases and that information on the topic is lacking. In my opinion it would make more sense to analyze the rate of bleeding in young adults with known AC and maybe analyze which patients are at risk for a bleeding. The comparison to cSDH patients does not add much information.
- The authors treat these patients by craniotomy, which is a rather aggressive approach. There is no literature showing that craniotomy, hematoma evacuation, and cyst resection/fenestration is superior to burr hole and treatment of the hematoma alone. An additional option would be burr hole hematoma fenestration and endoscopic cyst fenestration (which is nowadays the more minimal invasive approach to AC leading to good results as well).
- The conclusion that AC bleeding may be caused by minor trauma or sport activities is not supported by the literature, and also not something that can be concluded for the authors data. There are studies showing that sport is not contraindicated in these patients since the risk for bleeding is similar for patient with AC whether they do sport or not (even contact sport). The conclusion that these patients “must” be followed is not supported by the data presented, it is merely a subjective impression of the authors. If the authors had analyzed how many patients with AC have bled they could recommend whether follow up is indicated in these patients. Most of these patients do not bleed and therefore follow up is not a “must”. In addition, as described by the authors most patients show (mild) symptoms, meaning they should be informed that if symptoms arise they should seek medical attention, but not necessarily need to be followed routinely.
- The conclusions drawn by the authors are somewhat flawed. The methodology comparing CSDH with AC to other CSDH patients is not ideal as mentioned above. For these reasons I unfortunately do not recommend the indexing of this work.

Is the work clearly and accurately presented and does it cite the current literature?

No

If applicable, is the statistical analysis and its interpretation appropriate?

Partly

Are all the source data underlying the results available to ensure full reproducibility?

No

Is the study design appropriate and is the work technically sound?

No

Are the conclusions drawn adequately supported by the results?

No

Are sufficient details of methods and analysis provided to allow replication by others?

Yes

Reviewer Expertise:

Neurosurgery, Pediatric Neurosurgery, Endoscopy, Vaskular Neurosurgery

I confirm that I have read this submission and believe that I have an appropriate level of expertise to state that I do not consider it to be of an acceptable scientific standard, for reasons outlined above.

# Response

**Published:** 2022-01-16

**Authors:** Huseyin Berk Benek

**Affiliations:** Bozyaka Training and Research Hospital, Turkey

- *Although the authors claim they add to the literature with their 10 cases and that information on the topic is lacking. In my opinion it would make more sense to analyze the rate of bleeding in young adults with known AC and maybe analyze which patients are at risk for a bleeding. The comparison to cSDH patients does not add much information.*
- **Response:**
  - Most of the studies about this subject analyzed the complicated ACs, from a total of patients with cSDH. The references of our study indicate all the studies and reviews. Parsch *et al*. identified AC in 16 of 658 patients (2.4%) with cSDH. They stated that subdural hematomas are infrequently encountered complications of arachnoid cysts.
    - [2] Parsch CS, Krauss J, Hofmann E, Meixensberger J, Roosen K.Arachnoid cysts associated with subdural hematomas and hygromas: analysis of 16 cases, long-term follow-up, and review of the literature. Neurosurgery. 40 (1997), 483–490
    - [8] Takizawa K, Sorimachi T, Honda Y, Ishizaka H, Baba T, Osada T, et al. Chronic Subdural Hematomas Associated with Arachnoid Cysts: Significance in Young Patients with Chronic Subdural Hematomas. Neurol Med Chir (Tokyo). 55 (2015), 727-34.
  - Analyzing the rate of bleeding in young adults with known AC is really difficult, as most ACs do not have symptoms and do not undergo surgery. And it’s hard to find the proportion of ACs of the population  and the AC patients coming to outpatient clinics.
- *The authors treat these patients by craniotomy, which is a rather aggressive approach. There is no literature showing that craniotomy, hematoma evacuation, and cyst resection/fenestration is superior to burr hole and treatment of the hematoma alone. An additional option would be burr hole hematoma fenestration and endoscopic cyst fenestration (which is nowadays the more minimal invasive approach to AC leading to good results as well).*
- **Response:** Open craniotomy including the membranes of the AC and the cSDH removement, drainage of the hematoma using a burr hole, cyst fenestration or cystoperitoneal shunt could be chosen. The most common surgical method is open craniotomy alone. ^13,15^ Zuckermann *et al *stated that open craniotomy was the most common form of surgical treatment (%49). In their systematic review in Neurosurgical Focus , they analysed 65 cases in Table 4, and nearly half of all cases mentioned cyst fenestration after open craniotomy.
  - [13] Zuckerman SL, Prather CT, Yengo-Kahn AM, Solomon GS, Sills AK, Bonfield CM. Sport-related structural brain injury associated with arachnoid cysts: a systematic review and quantitative analysis. Neurosurg Focus. 40(4) (2016), E9
  - [15] Takayasu T, Harada K, Nishimura S, Onda J, Nishi T, Takagaki H. Chronic subdural hematoma associated with arachnoid cyst: two case histories with pathological observations. Neurol Med Chir (Tokyo). 52 (2012), 113-117
- *The conclusion that AC bleeding may be caused by minor trauma or sport activities is not supported by the literature, and also not something that can be concluded for the authors data. There are studies showing that sport is not contraindicated in these patients since the risk for bleeding is similar for patient with AC whether they do sport or not (even contact sport). The conclusion that these patients “must” be followed is not supported by the data presented, it is merely a subjective impression of the authors. If the authors had analyzed how many patients with AC have bled they could recommend whether follow up is indicated in these patients. Most of these patients do not bleed and therefore follow up is not a “must”. In addition, as described by the authors most patients show (mild) symptoms, meaning they should be informed that if symptoms arise they should seek medical attention, but not necessarily need to be followed routinely.*
- **Response:**
  - Several case reports of cSDH associated with ACs have been reported in the literature after head injury in sports. ^20,21,22,23^ Sport is an important factor of cSDHs in young patients.    
    - [20] Tsuzuki N, Katoh H, Ohtani N. Chronic subdural hematoma complicating arachnoid cyst secondary to soccer-related head injury: case report. Neurosurgery. 53 (2003), 242-243
    -  [21] Demetriades AK, McEvoy AW, Kitchen ND. Subdural haematoma associated with an arachnoid cyst after repetitive minor heading injury in ball games. Br J Sports Med. 38 (2004), E8
    -  [22] Kertmen H, Gürer B, Yilmaz ER, Sekerci Z. Chronic subdural hematoma associated with an arachnoid cyst in a juvenile taekwondo athlete: a case report and review of the literature. Pediatr Neurosurg. 48 (2012), 55-58
    -  [23] Prabhu VC, Bailes JE. Chronic subdural hematoma complicating arachnoid cyst secondary to soccer-related head injury: case report. Neurosurgery. 50 (2002), 195–197
  - Also Mori et al stated in a study in Journal of Neurotrauma that AC is a risk factor for chronic subdural hematoma in juveniles. We changed “must” to “should” as recommended.
    [4] Mori K, Yamamoto T, Horinaka N, Maeda M. Arachnoid cyst is a risk factor for chronic subdural hematoma in juveniles: twelve cases of chronic subdural hematoma associated with arachnoid cyst. J Neurotrauma. 19 (2002), 1017–1027 
- *The conclusions drawn by the authors are somewhat flawed. The methodology comparing CSDH with AC to other CSDH patients is not ideal as mentioned above. For these reasons I unfortunately do not recommend the indexing of this work.*
- **Response:** cSDH is a rare complication in patients with intracranial AC. In the case series of Wu et al., the incidence of AC-associated cSDH was 1.9% (5 of 266). Wu X, Li G, Zhao J, Zhu X, Zhang Y, Hou K. Arachnoid Cyst-Associated Chronic Subdural Hematoma: Report of 14 Cases and a Systematic Literature Review. World Neurosurg. 109 (2018), e118-e130. It would be better to do a search in literature of our references.

# Peer review: Reviewer response for version 1

**DOI:** 10.5256/f1000research.56569.r99264

**Published:** 2021-12-01

**Authors:** Roberto Colasanti

**Affiliations:** 1 Department of Neurosurgery, Azienda Ospedali Riuniti, Università Politecnica delle Marche, Ancona, Italy; 2 Department of Neurosurgery, Padua University Hospital, Padova, Italy

In this manuscript, the authors report a series of 10 patients with concomitant chronic subdural hematomas and arachnoid cysts.

- The authors could briefly summarize and underline the novelty of their findings.
- As the authors stated, various treatment modalities have been proposed for the management of patients with CSDH and arachnoid cysts (open craniotomy, burr hole and drainage of the hematoma, cyst fenestration or cystoperitoneal shunt). They could briefly report about the pros and cons of the different treatments as well as of the associated recurrence rates.
- Could embolization be helpful to increase the complete cure rate and avoid recurrences? What about medical therapy, e.g. corticosteroids?
- In the introduction section, the topic of the manuscript (concomitant chronic subdural hematomas and arachnoid cysts) could be better introduced. What about the incidence of CSDH in patients with arachnoid cysts?
- A clinical deterioration due to hemorrhage into the cyst or subdural compartment represents a typical presentation of an arachnoid cyst. I believe it is quite obvious that “presence of an arachnoid cyst in young adults is a predisposing factor for the formation of a chronic subdural hematoma with or without a head trauma”.
- English needs to be revised.

Is the work clearly and accurately presented and does it cite the current literature?

Partly

If applicable, is the statistical analysis and its interpretation appropriate?

Partly

Are all the source data underlying the results available to ensure full reproducibility?

Yes

Is the study design appropriate and is the work technically sound?

Partly

Are the conclusions drawn adequately supported by the results?

Partly

Are sufficient details of methods and analysis provided to allow replication by others?

Yes

Reviewer Expertise:

Neurosurgery

I confirm that I have read this submission and believe that I have an appropriate level of expertise to confirm that it is of an acceptable scientific standard, however I have significant reservations, as outlined above.

# Response

**Published:** 2021-12-02

**Authors:** Huseyin Berk Benek

**Affiliations:** Bozyaka Training and Research Hospital, Turkey

- *The authors could briefly summarize and underline the novelty of their findings.*
- **Response:** We accepted the suggestion and summarized it. Although there are several previous manuscripts, our study is one of the most comprehensive assessments of the combination of ACs and cSDH. Since there is still a gap of knowledge about this subject today, we tried to contribute to the body of literature.  
- *As the authors stated, various treatment modalities have been proposed for the management of patients with CSDH and arachnoid cysts (open craniotomy, burr hole and drainage of the hematoma, cyst fenestration or cystoperitoneal shunt). They could briefly report about the pros and the cons of the different treatments as well as the associated recurrence rates.*
- **Response:**
  - Complex dissection interventions could be necessary in such cases in which a burr hole is insufficient in the management. A single burr-hole-mini craniotomy and hematoma evacuation followed by endoscopic inspection of the surgical cavity could be a preferred choice. Initial observation may be considered in patients with a thickness of 10 mm or less cSDH diameter without symptoms of intracranial hypertension.
  - Recurrence can occur in 10%-20% of patients and is associated with several clinical and radiographic predictors. (Feghali J, Yang W, Huang J. Updates in Chronic Subdural Hematoma: Epidemiology, Etiology, Pathogenesis, Treatment, and Outcome. World Neurosurg. 2020 Sep;141:339-345). Postoperative CSDH volume and the Nakaguchi classification subtypes proved the most powerful predictors of recurrence and cure. (separated or trabecular subtypes and postoperative CSDH volume ≥35.0 mL)
- *Could embolization be helpful to increase the complete cure rate and avoid recurrences? What about medical therapy, e.g. corticosteroids?*
- **Response:** We mentioned about middle meningeal artery embolization and medical therapy, e.g. corticosteroids as recommended. Middle meningeal artery embolization may be represented as a minimally-invazive alternative to surgery for new or recurrent chronic subdural hematomas, or as prophylaxis to reduce the risk of recurrence after surgery.
  - [24] Link TW, Boddu S, Paine SM, Kamel H, Knopman J. Middle Meningeal Artery Embolization for Chronic Subdural Hematoma: A Series of 60 Cases. Neurosurgery. 85(6) (2019), 801-807
  - [25] Ban SP, Hwang G, Byoun HS, Kim T, Lee SU, Bang JS, Han JH, Kim CY, Kwon OK, Oh CW. Middle Meningeal Artery Embolization for Chronic Subdural Hematoma. Radiology. 286(3) (2018), 992-999
  - Some studies suggest that corticosteroids might be beneficial in the treatment of CSDH; however, there is a lack of well-designed trials that support or refute the use of corticosteroids in CSDH. 
  - [26] Berghauser Pont LM, Dirven CM, Dippel DW, Verweij BH, Dammers R. The role of corticosteroids in the management of chronic subdural hematoma: a systematic review. Eur J Neurol. 19(11) (2012), 1397-403
- *In the introduction section, the topic of the manuscript (concomitant chronic subdural hematomas and arachnoid cysts) could be better introduced. What about the incidence of CSDH in patients with arachnoid cysts?*
- **Response: **The introduction section has been revised as suggested. Chronic subdural hematoma (CSDH) is a complex disease with an overall incidence of 1.7-20.6 per 100,000 persons per year and is more commonly encountered in the elderly population. ACs are benign congenital lesions with a prevalence of 0.7% to 1,7% of the population. Since CSDH in arachnoid cysts patients is a rare phenomenon, several papers have been reported in the literature.
- *A clinical deterioration due to hemorrhage into the cyst or subdural compartment represents a typical presentation of an arachnoid cyst. I believe it is quite obvious that “ presence of an arachnoid cyst in young adults is a predisposing factor in the formation of a chronic subdural hematoma with or without a head trauma”.*
- **Response:** Previously reported rates of cSDH in young adults were 2.4–8.8% of all cSDH. Young adults with arachnoid cysts tend to be more susceptible to the development of chronic subdural hematomas.
- *English needs to be revised.*
- **Response: **As you suggested, we have revised our English draft once again. Also, our paper has been edited and certificated for English language and spelling by Enago (www.enago.com), an editing brand of Crimson Interactive Inc.

# Response

**Published:** 2021-12-05

**Authors:** Huseyin Berk Benek

**Affiliations:** Bozyaka Training and Research Hospital, Turkey

Additional response to the questions of reviewer 1:

- *What about the incidence of CSDH in patients with arachnoid cysts? A clinical deterioration due to hemorrhage into the cyst or subdural compartment represents a typical presentation of an arachnoid cyst. I believe it is quite obvious that “ presence of an arachnoid cyst in young adults is a predisposing factor the formation of a chronic subdural hematoma with or without a head trauma”.*
- **Response:**
  - cSDH is a rare complication in patients with intracranial AC. In the case series of Wu et al., the incidence of AC-associated cSDH was 1.9% (5 of 266).
  - [ Wu X, Li G, Zhao J, Zhu X, Zhang Y, Hou K. Arachnoid Cyst-Associated Chronic Subdural Hematoma: Report of 14 Cases and a Systematic Literature Review. World Neurosurg. 109 (2018), e118-e130]
  - Wester and Helland reported that 4.6% (11 of 241) of their patients admitted for AC were identified with cSDH. [Wester K, Helland CA.How often do chronic extra-cerebral haematomas occur in patients with intracranial arachnoid cysts? J Neurol Neurosurg Psychiatry. 79 (2008), 72–75]
  - Parsch et al. identified AC in 16 of 658 patients (2.4%) with cSDH. They stated that subdural hematomas are infrequently encountered complications of arachnoid cysts. [Parsch CS, Krauss J, Hofmann E, Meixensberger J, Roosen K.Arachnoid cysts associated with subdural hematomas and hygromas: analysis of 16 cases, long-term follow-up, and review of the literature. Neurosurgery. 40 (1997), 483–490]
